# Socioeconomic status and different forms of rhinitis in Swedish adults

**DOI:** 10.1002/clt2.12374

**Published:** 2024-06-19

**Authors:** Muwada Bashir Awad Bashir, Teet Pullerits, Linda Ekerljung, Helena Backman, Göran Wennergren, Hannu Kankaanranta, Bright I. Nwaru

**Affiliations:** ^1^ Krefting Research Centre Department of Internal Medicine and Clinical Nutrition Institute of Medicine, University of Gothenburg Gothenburg Sweden; ^2^ Krefting Research Centre University of Gothenburg Gothenburg Sweden; ^3^ Respiratory Medicine and Allergy Department of Internal Medicine & Clinical Nutrition Institute of Medicine University of Gothenburg Gothenburg Sweden; ^4^ Section of Allergology Sahlgrenska University Hospital Gothenburg Sweden; ^5^ Department of Public Health and Clinical Medicine Section of Sustainable Health/the OLIN Unit Umeå University Umeå Sweden; ^6^ Department of Pediatrics University of Gothenburg Gothenburg Sweden; ^7^ Department of Respiratory Medicine Seinäjoki Central Hospital Seinäjoki Finland; ^8^ Faculty of Medicine and Health Technology Tampere University Respiratory Research Group Tampere University Tampere Finland; ^9^ Wallenberg Centre for Molecular and Translational Medicine Institute of Medicine University of Gothenburg Gothenburg Sweden

**Keywords:** education, occupation, phenotypes, rhinitis, socioeconomic status

## Abstract

**Background:**

Rhinitis encompasses diverse forms. Each form has distinct pathophysiology and clinical manifestations and may be influenced by differential risk factors. The association between socioeconomic status (SES) and different forms of rhinitis remains poorly understood. Our aim was to examine SES variations in allergic rhinitis, chronic rhinitis, and chronic rhinosinusitis in adults.

**Methods:**

Based on a 2016 postal questionnaire survey within the West Sweden Asthma Study, we analyzed data from 36,213 subjects aged 16–75 years. The measures of SES were levels of education and occupation. Adjusted logistic regression was used to examine associations between SES and the rhinitis outcomes.

**Results:**

Attaining a secondary school and tertiary education, compared to a primary school, were associated with increased risk of allergic rhinitis (secondary OR 1.33, 95% CI 1.22–1.45; tertiary 1.54, 1.41–1.69) and chronic rhinitis (secondary 1.18, 1.08–1.29; tertiary 1.17, 1.06–1.28). The influence of occupation was consistent with respect to allergic rhinitis. For instance, compared to the lowest occupational skill level, the highest level (OR 1.24, 95% CI 1.04–1.48) and the lower high occupation levels (1.24, 1.04–1.49) were associated with an increased risk of allergic rhinitis. No significant link was found between education and chronic rhinosinusitis or between occupation levels and risk of either chronic rhinitis or chronic rhinosinusitis.

**Conclusion:**

Individuals with higher education and those at higher occupational levels may be at higher risk of having different forms of rhinitis than those at lower education and occupation levels. Assessment of rhinitis burden via SES can be one strategy to develop preventive strategies.

## 1 INTRODUCTION

Rhinitis is a heterogenous airway disorder encompassing various conditions characterized by symptoms arising from mucosal inflammation, including nasal congestion/obstruction, nasal pruritus, sneezing, and loss of smell.^1^ Different forms of rhinitis have been defined in the literature based on clinical presentation, etiology, and inflammatory profile.^2^ Allergic rhinitis, triggered by aeroallergens and mediated by immunoglobulin E, represents one such form.^3^ Chronic rhinitis is another form with symptoms lasting over 4 weeks, while rhinosinusitis, sharing symptoms with rhinitis, is characterized by two or more sino‐nasal symptoms such as congestion, discharge, facial pain, or loss of smell.^4^, ^5^ Chronic rhinosinusitis is a form, of the later, lasting more than 12 weeks.

Globally, allergic and non‐allergic rhinitis affect 20%–30% and 10%–15% of the population,^6^, ^7^ respectively, with increasing prevalence of allergic rhinitis in high income countries^8^ and both are significantly associated with respiratory diseases like asthma, common cold, and upper respiratory infections.^3^, ^5^, ^9^, ^10^, ^11^ Rhinitis exerts a considerable adverse impact on the quality of life by affecting sleep, work, school productivity, and psychological aspects.^12^, ^13^, ^14^

Precise diagnosis plays a critical role in effective management, and gaining insights into the risk factors associated with the forms of rhinitis is a valuable approach.^1^, ^2^ Both allergic and non‐allergic forms of rhinitis have been linked to environmental triggers in industrial settings and higher tobacco exposure in household setups.^15^, ^16^ There is also evidence suggesting that the risk of rhinitis may vary among socioeconomic groups. Lower socioeconomic status (SES) is associated with a reduced risk of allergic rhinitis,^17^ while family factors in high social status may increase the risk.^18^ The relationship between SES and chronic rhinosinusitis is mixed, as both higher and lower SES have been seen as risk factors, depending on the SES indicators used.^19^

The majority of previous studies have concentrated on the allergic form of rhinitis when exploring the role of SES. Therefore, there are limited data elucidating the role of SES in other forms of rhinitis. To fill this gap, the current study, based on data from a large population‐representative study of Swedish adults, aimed to investigate whether SES, based on education and occupation, is a determinant of the risk of different forms of rhinitis.

## 2 METHODS

### 2.1 Sample and population

**FIGURE 1:**
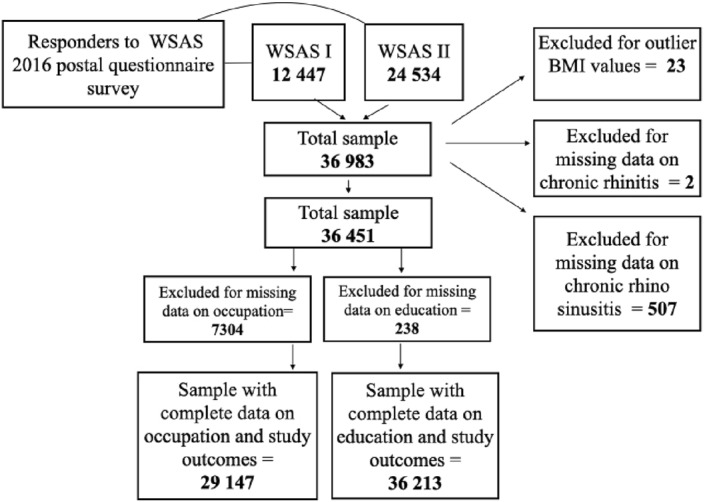
The study sample and number of participants from West Sweden Asthma Study (WSAS) 1 and WSAS 2.

The study sample was drawn from a population of adults living in Southwestern Sweden who participated in a postal questionnaire survey conducted in 2016 within the West Sweden Asthma Study (WSAS). WSAS is a large population‐representative study comprising adults and adolescents aged 15–75 years. The study was approved by the regional ethical review board in Gothenburg, Sweden. All participants gave their written informed consent to participate in the study by completing and returning the postal questionnaire. Of the 36,792 participants, 36,213 subjects had complete outcome and education data, while 29,147 subjects had complete outcome and occupation classification data (Figure 1). Detailed description of WSAS has been documented previously.^20^

### 2.2 Questionnaire

A validated questionnaire, originally developed following the structure of the British Medical Research Council Questionnaire and used in previous large‐scale studies,^21^, ^22^, ^23^, ^24^, ^25^7 was employed in this study. The questionnaire inquired about respiratory diseases, risk factors, treatment, as well as occupational and educational levels. For more details regarding the questionnaire utilized in this research, comprehensive information is available elsewhere.^20^

### 2.3 Definitions

#### 2.3.1 Exposures

**Education level:** level of education was measured by subjects' self‐reports of highest attained education, that is, primary education, secondary education, or tertiary education.

**Occupational Skill Levels:** these were determined by applying the Swedish Standard Classification of Occupations (SSYK),^26^ which is aligned with the International Standard Classification of Occupations 2012 (ISCO‐12).^27^ The various occupations were categorized into four distinct skill levels: highest, lower high, upper low, and lowest. Each of these skill levels corresponds to the educational qualifications necessary for job attainment.

#### 2.3.2 Outcomes

**Allergic rhinitis**: self‐report of positive answers to the question “Have you ever had allergic eye or nose problems (hay fever)?” from a postal survey questionnaire.

**Chronic rhinitis:** self‐report of positive answers to the question “Have you had any rhinitis and either of nasal blockage more or less constantly or having rhinitis more or less constantly” from a postal survey questionnaire.

**Chronic rhinosinusitis:** self‐report of positive answer to three or more of the following questions from postal survey questionnaire:

‐ Have you had a stuffy nose for more than 12 weeks in the last 12 months?
‐ Have you had pain or pressure around your forehead, nose, or eyes for more than 12 weeks in the last 12 months?
‐ Have you had discolored nasal secretions (strings) or discolored mucus in your throat for more than 12 weeks in the last 12 months?
‐ Has your sense of smell been impaired or gone for more than 12 weeks in the last 12 months?

### 2.4 Statistical analysis

For descriptive analysis, we performed the Pearson chi‐square test to examine differences in distribution of covariates by categorical groups of exposures. We also examined the variation in the prevalence of each form of rhinitis by SES groups within strata of age groups, sex, body mass index (BMI) levels, and family history of asthma or allergy and whether they were raised on a farm or not. We employed logistic regression to model the associations between SES and each form of rhinitis. Each model was adjusted for age, sex, BMI, family history of allergy or asthma, smoking status, smoking exposure at home or work, and exposure to vapor, gas, dust and fumes (VGDF) at work, being raised on a farm, or being raised in rural areas as a child. We estimated the associations between SES and outcomes using odds ratios and their corresponding 95% confidence interval (CI). We reported *p*‐values for the comparison of the unadjusted model with exposure only versus the adjusted model including the full list of covariates, with separate models for education and occupation.

#### 2.4.1 Covariates

Data on covariates, such as age, sex, BMI, family history of allergy or asthma, smoking status, smoking exposure at home and work, and exposure to VGDF at work, growing up on a farm during childhood, or living in a rural area during childhood, were collected.

#### 2.4.2 Interaction and moderation analysis

We assessed whether education and occupation exhibited interaction effects with covariates in relation to each form of rhinitis. We conducted model comparisons, contrasting a model incorporating interaction terms between SES and each covariate with one lacking such terms. In cases where the difference in model coefficients reached significance, we conducted further analyses comparing the simple effects of each interacting factor across exposure groups and vice versa. To mitigate the risk of obtaining statistically significant results by chance alone, we applied the Bonferroni adjustment method during this later step as recommended.^28^ Our presentation of the interaction analysis results included odds ratios and their 95% confidence intervals. Additionally, we visually depicted the interaction effect through graphical representation using predicted probabilities derived from the logarithms of estimated values along with 95% confidence intervals.

## 3 RESULTS

### 3.1 Characteristics of the participants

The majority of participants were aged 30 or older, with more females than males. Among the participants, 14% were classified as obese, approximately 36% were overweight, and the majority fell within the normal weight range. About one‐third of the sample reported a family history of allergy or asthma (35%), and a similar proportion reported having lived in a rural area during childhood. Approximately 12% of the participants indicated being raised on a farm during their childhood. Regarding smoking habits, around 25% of participants were former smokers, while 11% were current smokers. Sixteen percent of the sample reported exposure to smoking at home, and 9% reported exposure to smoking at work (Table 1).

**TABLE 1:** Characteristics of study sample by education levels.

	Primary	Secondary	Tertiary	Total	*p* Value
*n*	5659	14,243	16,311	36,213	
Age group (%)					<0.001
Less than 30	436 (7.7)	2578 (18.1)	2814 (17.3)	5828 (16.1)	
30–45	469 (8.3)	3271 (23.0)	5366 (32.9)	9106 (25.1)	
46–60	1527 (27.0)	4544 (31.9)	4605 (28.2)	10,676 (29.5)	
≥61	3227 (57.0)	3850 (27.0)	3526 (21.6)	10,603 (29.3)	
Sex					<0.001
Females (%)	2766 (48.9)	7301 (51.3)	9844 (60.4)	19,911 (55.0)	
Males (%)	2893 (51.1)	6942 (48.7	6467 (39.6)	16,302 (45.0)	
BMI (%)					<0.001
Normal weight	2087 (36.9)	6382 (44.8)	9131 (56.0)	17,600 (48.6)	
Overweight	2260 (39.9)	5350 (37.6)	5256 (32.2)	12,866 (35.5)	
Obese	1139 (20.1)	2211 (15.5)	1653 (10.1)	5003 (13.8)	
Missing	173 (3.1)	300 (2.1)	271 (1.7)	744 (2.1)	
Family history of allergy or asthma (%)					<0.001
Yes	1439 (25.4)	4841 (34.0)	6287 (38.5)	12,567 (34.7)	
Missing	393 (6.9)	523 (3.7)	357 (2.2)	1273 (3.5)	
Smoking status (%)					<0.001
Never smoker	2949 (52.1)	8456 (59.4)	11,409 (69.9)	22,814 (63.0)	
Former smoker	1760 (31.1)	3719 (26.1)	3613 (22.2)	9092 (25.1)	
Current smoker	891 (15.7)	1966 (13.8)	1207 (7.4)	4064 (11.2)	
Missing	59 (1.0)	102 (0.7)	82 (0.5)	243 (0.7)	
Smoking exposure at home (%)					<0.001
Yes	1142 (20.2)	2609 (18.3)	2353 (14.4)	6104 (16.9)	
Missing	124 (2.2)	227 (1.6)	207 (1.3)	558 (1.5)	
Smoking exposure at work (%)					<0.001
Yes	857 (15.1)	1665 (11.7)	958 (5.9)	3480 (9.6)	
Missing	119 (2.1)	216 (1.5)	200 (1.2)	535 (1.5)	
VGDF exposure at work (%)					<0.001
Yes	1718 (30.4)	3401 (23.9)	1452 (8.9)	6571 (18.1)	
Missing	188 (3.3)	215 (1.5)	227 (1.4)	630 (1.7)	
Raised on a farm during childhood (%)					<0.001
Yes	1366 (24.1)	1733 (12.2)	1428 (8.8)	4527 (12.5)	
Missing	98 (1.7)	210 (1.5)	265 (1.6)	573 (1.6)	
Rural residence during childhood (%)					<0.001
Yes	2699 (47.7)	5125 (36.0)	4718 (28.9)	12,542 (34.6)	
Missing	92 (1.6)	157 (1.1)	214 (1.3)	463 (1.3)	

Abbreviation: BMI, body mass index.

Individuals with primary education were older (57%), more males, and were less often obese (20%) than those with secondary and tertiary education. Additionally, those with primary education were more likely to have no family history of allergy or asthma (67%), more often never smokers (52%), and less likely to be exposed to smoking at home or VGDF at work than those with secondary and tertiary education levels (Table 1).

Individuals in both the lowest and upper low occupational skill categories were more often between 30 and 60 years of age than in other occupational skill groups. They also constituted more females than males and were normal to overweight and had a lower proportion of family history of allergy or asthma than other occupational skill groups. The two lowest occupational skill groups had more never smokers and less individuals with exposure to smoking at home or work or to VGDF at work than other occupational skill groups (Table 2).

**TABLE 2:** Characteristics of study sample by occupation levels.

	Lowest	Upper low	Lower high	Highest	Total	*p* Value
*n*	1072	14,240	5191	8644	29,147	
Age group (%)						<0.001
Less than 30	237 (22.1)	1834 (12.9)	625 (12.0)	1088 (12.6)	3784 (13.0)	
30–45	312 (29.1)	3619 (25.4)	1612 (31.1)	2843 (32.9)	8386 (28.8)	
46–60	310 (28.9)	4999 (35.1)	1685 (32.5)	2596 (30.0)	9590 (32.9)	
≥61	213 (19.9)	3788 (26.6)	1269 (24.4)	2117 (24.5)	7387 (25.3)	
Sex						<0.001
Females (%)	724 (67.5)	7648 (53.7)	2268 (43.7)	5333 (61.7)	15,973 (54.8)	
Males (%)	348 (32.5)	6592 (46.3)	2923 (56.3)	3311 (38.3)	13,174 (45.2)	
BMI (%)						<0.001
Normal weight	482 (45.0)	6177 (43.4)	2586 (49.8)	4853 (56.1)	14,098 (48.4)	
Overweight	372 (34.7)	5478 (38.5)	1909 (36.8)	2824 (32.7)	10,583 (36.3)	Overweight
Obese	189 (17.6)	2355 (16.5)	629 (12.1)	835 (9.7)	4008 (13.8)	
Missing	29 (2.7)	230 (1.6)	67 (1.3)	132 (1.5)	458 (1.6)	
Family history of allergy or asthma (%)						<0.001
Yes	387 (36.1)	4927 (34.6)	1758 (33.9)	3284 (38.0)	10,356 (35.5)	
Missing	62 (5.8)	563 (4.0)	98 (1.9)	166 (1.9)	889 (3.1)	
Smoking status (%)						<0.001
Never smoker	580 (54.1)	8093 (56.8)	3553 (68.4)	6079 (70.3)	18,305 (62.8)	
Former smoker	253 (23.6)	4076 (28.6)	1212 (23.3)	1997 (23.1)	7538 (25.9)	
Current smoker	229 (21.4)	2006 (14.1)	411 (7.9)	542 (6.3)	3188 (10.9)	
Missing	10 (0.9)	65 (0.5)	15 (0.3)	26 (0.3)	116 (0.4)	
Smoking exposure at home (%)						<0.001
Yes	216 (20.1)	2775 (19.5)	757 (14.6)	1225 (14.2)	4973 (17.1)	
Missing	22 (2.1)	187 (1.3)	55 (1.1)	75 (0.9)	339 (1.2)	
Smoking exposure at work (%)						<0.001
Yes	179 (16.7)	1750 (12.3)	394 (7.6)	391 (4.5)	2714 (9.3)	
Missing	19 (1.8)	164 (1.2)	48 (0.9)	78 (0.9)	309 (1.1)	
VGDF exposure at work (%)						<0.001
Yes	345 (32.2)	4105 (28.8)	579 (11.2)	499 (5.8)	5528 (19.0)	
Missing	23 (2.1)	156 (1.1)	48 (0.9)	98 (1.1)	325 (1.1)	
Raised on a farm during childhood (%)						<0.001
Yes	171 (16.0)	2160 (15.2)	469 (9.0)	769 (8.9)	3569 (12.2)	
Missing	24 (2.2)	154 (1.1)	60 (1.2)	116 (1.3)	354 (1.2)	
Rural residence during childhood (%)						<0.001
Yes	395 (36.8)	5615 (39.4)	1638 (31.6)	2509 (29.0)	10,157 (34.8)	
Missing	20 (1.9)	114 (0.8)	44 (0.8)	88 (1.0)	266 (0.9)	

Abbreviation: BMI, body mass index.

### 3.2 Prevalence of forms of rhinitis by SES indicators

The overall prevalence of allergic rhinitis was 28% (95% CI: 27.8%–28.8%), chronic rhinitis 19% (95% CI: 19%–20%), and chronic rhinosinusitis 2.7% (95% CI: 2.6%–2.8%).

#### 3.2.1 Education

**FIGURE 2:**
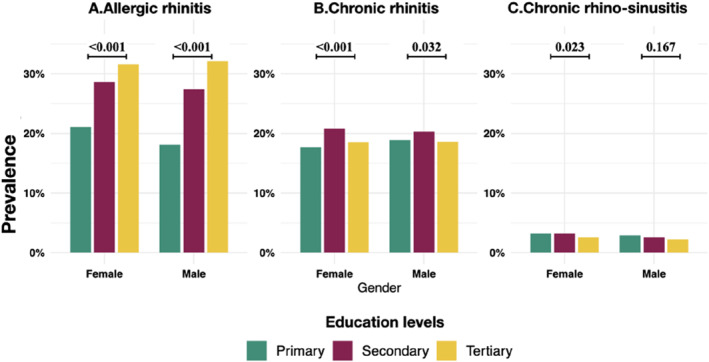
Prevalence of forms of rhinitis by education levels among males and females with *p* value for between education group comparison.

A higher prevalence of allergic rhinitis was seen among those with secondary and tertiary education than among those with primary education, both among males and females (Figure 2). Within males, chronic rhinitis was more common among secondary education, but less common among those with tertiary education than among those with primary education. However, a higher prevalence of chronic rhinitis among secondary and tertiary education than primary education was observed among females. Among both males and females, chronic rhinitis was higher among secondary than primary education. Chronic rhinosinusitis was higher among those with primary and secondary education than among those with tertiary education among females, but no difference in chronic rhinosinusitis by education was found among males (Figure 2).

#### 3.2.2 Occupation

**FIGURE 3:**
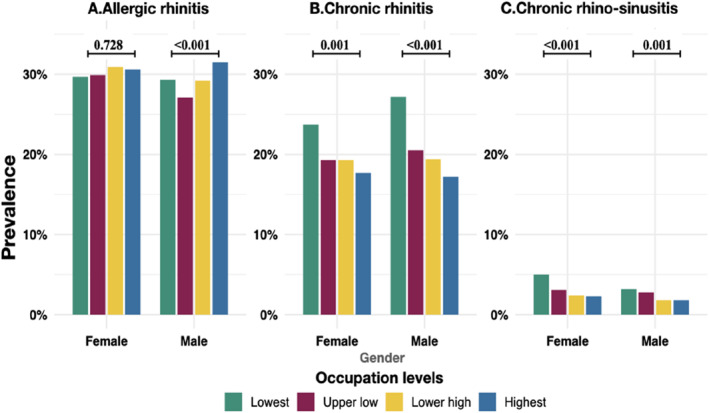
Prevalence of forms of rhinitis by occupation levels among males and females with *p* value for comparison between occupation groups.

Within sex strata, there was variation by occupation groups among males but not among females. The prevalence of allergic rhinitis was higher among the two highest occupation levels than among the lowest levels among males but not females (Figure 3A). Chronic rhinitis was less common among higher occupation skill levels than among the lowest levels. However, the prevalence of chronic rhinosinusitis was lower among high occupational skill levels than the lowest skill groups, both among males and females (Figure 3B,C).

**FIGURE 4:**
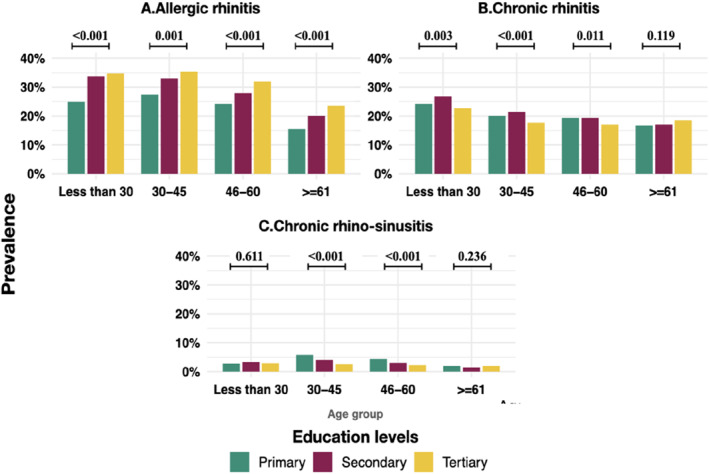
Prevalence of forms of rhinitis by education levels among age groups with the *p* value for comparison between education groups.

Within age strata, the prevalence of allergic rhinitis was higher among higher education and higher occupation groups than in the lower groups (Figure 4A). Chronic rhinitis was lower among tertiary educated compared with primary educated among those aging less than 60 years (Figure 4B). Chronic rhinosinusitis was lower among higher than lower education groups among those aged between 30 and 60 years. (Figure 4C in the main text and Tables S1 and S2).

**FIGURE 5:**
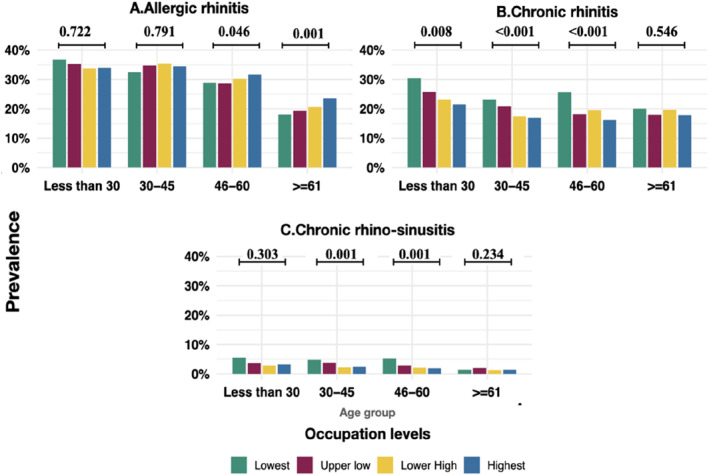
Prevalence of forms of rhinitis by occupation levels among age groups the *p* value for between occupation group comparison.

The prevalence of allergic rhinitis was higher among the highest occupation levels compared with the lowest occupation only among the elderly (Figure 5A). Chronic rhinosinusitis was less prevalent among highest occupation levels than lowest among those aging less than 60 years (Figure 5B), while chronic rhinosinusitis was lower among highest occupations than lowest among those aging between 30 and 60 years (Figure 5C).

With regard to being raised on a farm or not, the prevalence of allergic rhinitis was higher among higher than lower education groups among those who were and were not raised on a farm but did not vary by occupation groups. Chronic rhinitis was also higher among higher than lower education groups among the two strata. However, chronic rhinitis was more prevalent among the lowest occupation than among the highest occupation among those who were not raised on a farm. Chronic rhinosinusitis was more prevalent among the lowest than highest education levels only among those who were not raised on a farm. Similarly, lowest occupation levels had a higher prevalence of chronic rhinosinusitis among both strata of being raised on a farm (Table S3).

### 3.3 Association between SES and forms of rhinitis

#### 3.3.1 Education

**FIGURE 6:**
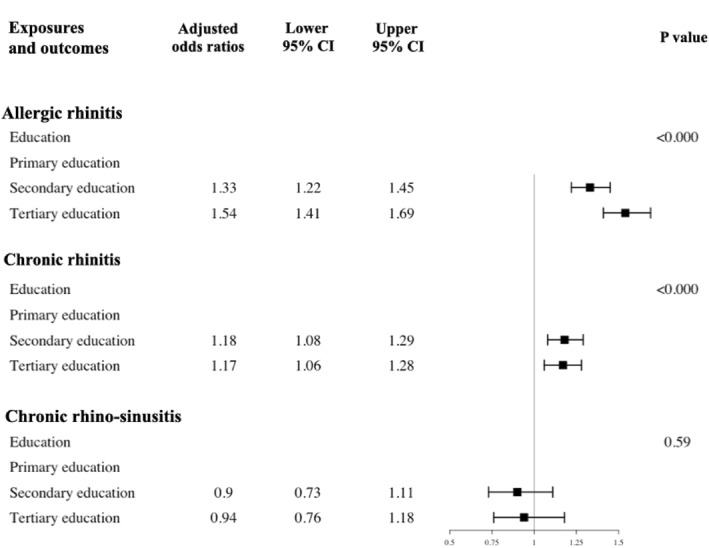
The odds ratios and their 95% confidence intervals for the association between education and forms of rhinitis.

After adjusting for covariates, compared to primary education, secondary and tertiary education were associated with an increased risk of allergic and chronic rhinitis, but not chronic rhinosinusitis (Figure 6).

#### 3.3.2 Occupation

**FIGURE 7:**
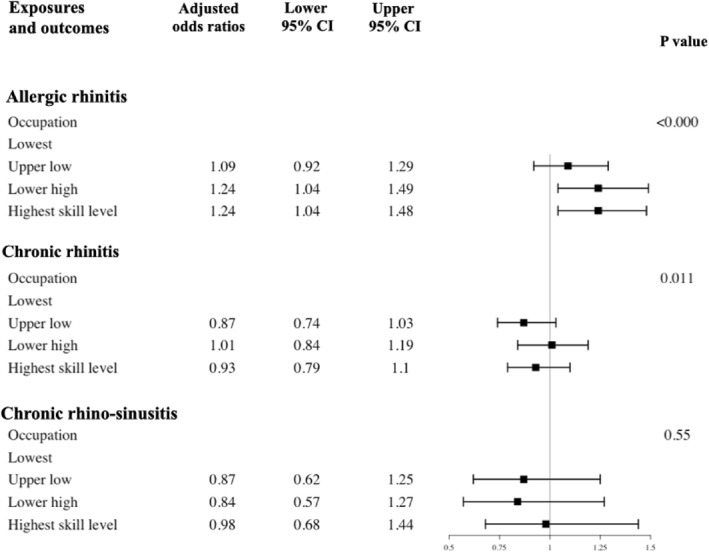
The odds ratios and their 95% confidence intervals for the association between occupation, and forms of rhinitis.

After adjustment for covariates, compared to the lowest occupational skill group, those in the two highest occupational skill groups were at an increased risk of having allergic rhinitis. There was no association between occupational skill groups and chronic rhinitis or chronic rhinosinusitis (Figure 7).

For sensitivity analysis, we also tested for the association among samples stratified by asthma status and whether subjects were brought up in rural or urban settings. By stratifying the analyses by presence of asthma, the results were similar between those who had asthma and those without asthma such that those with secondary and tertiary education, compared to those with primary education, were at an increased risk of having allergic rhinitis. While no putative associations were observed between educational levels and chronic rhinitis among those with asthma, among non asthma subjects, those with secondary and tertiary education, compared with those with primary education, were at an increased risk of having chronic rhinitis. Educational levels were not associated with chronic rhinosinusitis and occupational levels were not associated with any form of rhinitis in the asthma‐stratified analysis. By stratifying the analyses by urban versus rural childhood residence, the results were similar between those who resided in urban and those who resided in rural environments during childhood regarding the association between educational levels and allergic and chronic rhinitis. Educational levels were not associated with chronic rhinosinusitis, nor were occupational levels associated with any form of rhinitis in the analyses stratified by urban versus rural childhood residence. Full information in (Tables S4 and S5).

### 3.4 Interaction between socioeconomic status and subjects' characteristics in relation to the risk of forms of rhinitis

There were some interactions between education and each of age, sex and BMI in relation to the risk of some forms of rhinitis. For example, for allergic rhinitis, the increased risk observed for tertiary education compared to primary school was higher among males (OR 2.03, 95%CI 1.92–2.14) than females (OR 1.56, 95%CI 1.37–1.76) (Table S6 and figure S1).

For chronic rhinitis, the increased risk observed for tertiary compared to primary education was only evident among those older than 60 years (OR 1.38, 95%CI 1.21–1.58) and those aged 46–60 years (OR 1.16, 95%CI 1.00–1.36), but not among those aged 30–45 years (OR 1.01, 95%CI 0.76–1.36) and those less than 30 years of age (OR 0.92, 95%CI 0.74–1.16) (Table S4 and Figure S1). Finally, the increased risk observed for tertiary compared to primary school in relation to chronic rhinitis was only evident among those who were obese (OR 1.22, 95%CI 1.00–1.42) and overweight (OR 1.32, 95%CI 1.15–1.52) but not among those with normal weight (OR 1.03, 95%CI 0.95–1.11) (Table S7 and Figure S1).

## 4 DISCUSSION

In this large population‐representative sample of adults, individuals with secondary or tertiary education attainment were more likely to report the presence of allergic or chronic forms of rhinitis than individuals with primary education. Furthermore, individuals in the higher occupational skill groups were more likely to report the presence of allergic rhinitis than those in the lowest occupation levels. No difference was observed by education levels with respect to the risk of chronic rhinosinusitis, while no difference was observed by occupation skill levels with respect to the risk of either chronic rhinitis or chronic rhinosinusitis. Although not a consistent finding, there was some evidence that sex, age and BMI may modify these associations.

WSAS, characterized by its large sample size and random selection of participants, is reliably representative of the adult population of southwestern Sweden. The data collection process employed standard questionnaires validated in prior studies.^21^, ^22^, ^23^, ^24^, ^25^ The robust combination of a substantial and randomly chosen sample coupled with high‐quality data increases the reliability and validity of our findings.

Unlike prior investigations that primarily investigated the association between SES and allergic rhinitis,^29^ the inclusion of several rhinitis forms in our study provides a more comprehensive examination of SES variations in the prevalence of rhinitis. Furthermore, the inclusion of both education and occupation as indicators of SES provides further insights into this study topic.

The cross‐sectional design of the current study means that we cannot infer whether SES status predisposed individuals to rhinitis risk or the other way round. Therefore, the lack of temporality in the investigated associations is a limitation of the current study. In addition, the collection of the study variables solely on self‐report of participants increased the chance for information bias. Commonly, accurate diagnosis of rhinitis, particularly based on individuals' report, can be challenging as self‐awareness and manifestation of symptoms similar to the conditions, for example, common cold, can cause confusion. Nevertheless, the assessment of individuals' self‐report or self‐perception of respiratory conditions has been recognized as an important aspect of a broader understanding of the burden of these diseases. Our generally adopted approach for defining rhinitis phenotypes based on self‐report of having nasal symptoms of sneezing, or a runny, or a blocked nose is in line with the recommended approaches suggested by the International Study of Asthma and Allergies in Childhood,^30^ as recommended by Allergic Rhinitis and its Impact on Asthma (ARIA).^31^

Although education and occupation status are widely accepted SES measures in epidemiological literature, their impact on outcomes influenced by factors such as air pollution, occupational exposures, and household conditions may vary. To enhance accuracy, incorporating objective measures of occupation exposures, such as a job occupation matrix, would have increased the reliability and validity of these SES measures. This approach may help to mitigate bias introduced by factors such as changing jobs due to health issues, job selection based on health status, and the clustering of health problems among certain occupations.

Our finding of increased risk of allergic rhinitis with higher than lower education and skill occupation levels is consistent with findings from Bråbäck et al,^17^ which defined SES based on education, occupation, type of production and the position at work. Mercer et al.^32^ also found an increased risk of allergic rhinitis from areas with the lowest to the highest SES based on residential status. Furthermore, Torfi et al.^33^ reported a consistent pattern of higher risk of allergic rhinitis with higher SES based on family income. However, in the same study, consideration was made for paternal and maternal SES based levels of education and found that a lower paternal education level was associated with an increased risk of allergic rhinitis. In their work among children, Biagini et al.^16^ reported that SES based on household conditions was not associated with an increased risk of allergic rhinitis.

Our finding of higher risk of chronic rhinitis among individuals with secondary and tertiary education attainment, compared to primary education, was hypothetically in line with reports in previous studies that found high risk of chronic rhinitis among smokers in occupations exposed to fumes dust and gases.^34^ Another study reported a higher risk of chronic rhinitis that is associated with higher exposure to particulate matter and inhalable particulate matter like Sulfur dioxide, nitric oxide, and carbon monoxide.^35^ Despite the unobserved association between risk of chronic rhinitis and occupational classes per se in our study, our finding of higher risk of chronic rhinitis in association with higher education classes remains conceivably surprising and justifies further investigation.

Our study did not capture an association between education and occupation classification as indicators of SES and risk of chronic rhinosinusitis. However, in previous reports, individuals with a high level of education attainment were at a decreased risk of chronic rhinosinusitis compared with those with a low level of education.^36^, ^37^, ^38^ Regarding occupation, some previous studies have reported results of increased risk of chronic rhinosinusitis among workers in low skill occupational groups, such as manual jobs, plant or machine operators and assemblers, elementary occupations, and craft and related trade workers, compared with workers in high skill occupational groups.^39^, ^40^

The observed association between a higher social class, as indicated by both occupation and education levels, and an increased risk of allergic rhinitis aligns with the hygiene theory. Elevated levels of education and engagement in high‐skill occupations may signify an improved material capital status, better household conditions, and potentially greater intellectual assets. These factors contribute to maintaining a hygienic living environment, which may consequently increase susceptibility to atopic conditions and allergic rhinitis. This theory suggests a heightened susceptibility to allergic conditions in individuals who have been raised in, and experiencing, cleaner environments.^41^ Another plausible explanation of the observed association between higher SES and risk or allergic rhinitis and chronic rhinitis might be the higher disease awareness that is a motive for seeking medical care and getting diagnosed and hence higher reporting of the condition among subjects with higher education and occupation levels than those with lower levels.^42^ Such hypothesis is further endorsed by the perceived higher accessibility to medical services by highly educated and occupational professionals due to higher income status.^43^ With respect to chronic rhinitis, our findings of higher risk among higher occupation and education levels can also be explained by hypothesized nasal irritation induced by exposure to ambient pollutants in urban settings.^44^ Further, the syndrome of nasal irritation and allergy is known to be associated with stress and anxiety.^45^ In the context of higher education and occupation, chronic stress might present as a proxy for demanding working conditions and long working hours, which in turn could aggravate symptoms of nasal inflammation.^46^

Education and occupation status are considered reliable indicators of social status as occupation often depends on the corresponding educational level. Our findings consistently show similar results between these two measures and allergic rhinitis. However, the observed discrepancy in the association between the two measures with respect to chronic rhinitis highlights the complexity of the effects of SES measures on various forms of rhinitis.

In this context, occupation relies on the cognitive capital of education rather than necessarily indicating a consistent material capital among individuals with high education and high occupation.^47^ This distinction may further manifest in various social exposures, such as household conditions, residential area exposures, and psychological quality of life, providing insights into the higher risk of chronic rhinitis among those with high education but no risk among individuals with high occupational skills.^43^, ^47^

Although our study did not capture the impact of education or occupation levels as indicators of SES, it is important to note that this result does not negate the possibility of other interconnected pathways through which socioeconomic exposures, which may not be adequately reflected in our measurement system, could influence the risk of chronic rhinosinusitis. In previous reports, high molecular weight agents such as flour, latex and laboratory animals as well as specific molecular weight agents were associated with a high risk of chronic rhinosinusitis in occupational settings.^48^, ^49^ Surprisingly, even workers in occupations traditionally considered less susceptible to such agents have demonstrated benefits from interventions such as thorough dust cleaning, resulting in a reduced risk of chronic rhinosinusitis.^44^ Therefore, additional research into the exposures within occupational settings and their potential association with the risk of chronic rhinosinusitis is warranted.

Our observations show that variables such as sex, age, and BMI may modify the association between SES and forms of rhinitis. In the case of sex, for instance, the modification may result from the distinct male‐female working conditions, their biological compositions, and hormonal aspects. All these aspects may combine to manifest in the observed sex‐differentiated association between SES and rhinitis. Regarding age‐modified associations between occupational skill groups and rhinitis phenotypes, the observed higher risk of chronic rhinitis among younger age groups may imply exposure to different working or environmental conditions, which places them at an elevated risk of developing chronic rhinitis. These groups may also exhibit distinct housing or neighborhood conditions that contribute to a heightened risk of experiencing allergic rhinitis or chronic rhinosinusitis.

Mechanistically, there may be a complex interplay between SES and environmental exposures in influencing the risk of different forms of rhinitis. Future research efforts should be directed towards gaining a deeper understanding of the various forms of rhinitis, considering aspects of pathophysiological mechanisms, chronicity, and comorbid presentations, and how SES influences these aspects. While education and occupation are reliable measures of SES, further granularity, and integration of other aspects of the socio‐environmental context are necessary when examining the role of SES towards forms of rhinitis. A more nuanced understanding of the underlying social components, such as housing, traffic, childhood, and family factors, is crucial in this respect and when designing effective management strategies.

## 5 CONCLUSION

Individuals with higher education and those at higher occupational levels may be at higher risk of having different forms of rhinitis than those at lower education and occupation levels. Assessment of rhinitis burden via SES can be one strategy to develop preventive strategies.

## AUTHOR CONTRIBUTIONS

**Muwada Bashir**: Data curation, formal analysis, methodology, project administration, software, validation, visualization, writing – original draft, writing – review & editing. **Teet Pullerits**: Methodology, project administration, resources, writing – review & editing. **Linda Ekerljung**: Conceptualization, funding acquisition, project administration, supervision, writing – review & editing. **Helena Backman**: Conceptualization, funding acquisition, methodology, validation, writing – review & editing. **Goran Wennergren**: Conceptualization; funding acquisition; project administration; supervision; writing – review & editing. **Hannu Kankaanranta**: Conceptualization, funding acquisition, methodology, supervision, writing – review & editing. **Bright Nwaru**: Conceptualization; funding acquisition; methodology; project administration; resources; supervision; writing – review & editing.

## CONFLICT OF INTEREST STATEMENT

HK reports personal fees for lectures and consulting from AstraZeneca, Boehringer‐Ingelheim, Chiesi, COVIS Pharma, GSK, Medscape, MSD, Novartis, Orion Pharma and Sanofi. All other authors of this work declare no conflicts of interest.

## Supporting information

Supporting Information S1

## Data Availability

Data sharing is not applicable to this article as no new data were created or analyzed in this study.
