# Supplementary material for: Socioeconomic status and different forms of rhinitis in Swedish adults
Source: Clin Transl Allergy. 2024 Jun 19;14(6):e12374. doi: 10.1002/clt2.12374 (PMC11186741; doi:10.1002/clt2.12374)
Supplement: Supplementary file 1 — Supporting Information S1 [file CLT2-14-e12374-s001.docx]

**Supplementary material**

Table S1. Prevalence of forms of rhinitis by education and occupation levels within male and females

|  | Male | | | | | | | Female | | | | | | |  |  |  |  |
| --- | --- | --- | --- | --- | --- | --- | --- | --- | --- | --- | --- | --- | --- | --- | --- | --- | --- | --- |
|  | **Primary** | | **Secondary** | | **Tertiary** | | **P**  **value** | | | **Primary** | | **Secondary** | | **Tertiary** | | **P**  **value** | | |
| n | 2880 | | 6928 | | 6457 | |  | | | 2746 | | 7281 | | 9821 | |  | | |
| Allergic rhinitis | 522 (18.1) | | 1901 (27.4) | | 2070 (32.1) | | <0.001 | | | 579 (21.1) | | 2081 (28.6) | | 3107 (31.6) | | <0.001 | | |
| Chronic rhinitis | 543 (18.9) | | 1409 (20.3) | | 1203 (18.6) | | 0.032 | | | 486 (17.7) | | 1513 (20.8) | | 1819 (18.5) | | <0.001 | | |
| Chronic rhinosinusitis | 83 ( 2.9) | | 178 ( 2.6) | | 145 ( 2.2) | | 0.167 | | | 88 ( 3.2) | | 235 ( 3.2) | | 252 ( 2.6) | | 0.023 | | |
|  | **Male** | | | | | | | | **Female** | | | | | | | | |  |
|  | **Lowest** | **Upper low** | | **lower high** | | **Highest** | | **p value** | **Lowest** | | **Upper low** | | **lower high** | | **Highest** | | **p Value** |  |
| n | 345 | 6576 | | 2921 | | 3308 | |  | 721 | | 7629 | | 2265 | | 5323 | |  |  |
| Allergic rhinitis | 101 (29.3) | 1782 (27.1) | | 852 (29.2) | | 1041 (31.5) | | <0.001 | 214 (29.7) | | 2281 (29.9) | | 699 (30.9) | | 1630 (30.6) | | 0.728 |  |
| Chronic rhinitis | 94 (27.2) | 1348 (20.5) | | 566 (19.4) | | 570 (17.2) | | <0.001 | 171 (23.7) | | 1470 (19.3) | | 437 (19.3) | | 940 (17.7) | | 0.001 |  |
| Chronic rhinosinusitis | 11  ( 3.2) | 187  ( 2.8) | | 53  ( 1.8) | | 60  ( 1.8) | | 0.001 | 36  ( 5.0) | | 237  ( 3.1) | | 54  ( 2.4) | | 124  ( 2.3) | | <0.001 |  |

Table S2. Prevalence of forms of rhinitis by education and occupation groups within strata of age groups and their p values

|  | Less than 30 | | | | | | | 30- 45 | | | | | | |  |  |  |  |
| --- | --- | --- | --- | --- | --- | --- | --- | --- | --- | --- | --- | --- | --- | --- | --- | --- | --- | --- |
|  | **Primary** | | **Secondary** | | **Tertiart** | | **P value** | | | **Primary** | | **Secondary** | | **Tertiart** | | **P value** | | |
| n | 434 | | 2571 | | 2810 | |  | | | 468 | | 3265 | | 5358 | |  | | |
| Allergic rhinitis | 108 (24.9) | | 867 (33.7) | | 979 (34.8) | | <0.001 | | | 128 (27.4) | | 1078 (33.0) | | 1896 (35.4) | | 0.001 | | |
| Chronic rhinitis | 105 (24.2) | | 689 (26.8) | | 639 (22.7) | | 0.003 | | | 94 (20.1) | | 698 (21.4) | | 948 (17.7) | | <0.001 | | |
| Chronic rhinosinusitis | 12 ( 2.8) | | 86 ( 3.3) | | 82 ( 2.9) | | 0.611 | | | 27 ( 5.8) | | 132 ( 4.0) | | 140 ( 2.6) | | <0.001 | | |
|  | **46 - 60** | | | | | | | | | **>=61** | | | | | | | | |
|  | **Primary** | | **Secondary** | | **Tertiart** | | **P value** | | | **Primary** | | **Secondary** | | **Tertiart** | | **P value** | | |
| n | 1520 | | 4536 | | 4598 | |  | | | 3204 | | 3837 | | 3512 | |  | | |
| Allergic rhinitis | 368 (24.2) | | 1266 (27.9) | | 1472 (32.0) | | <0.001 | | | 497 (15.5) | | 771 (20.1) | | 830 (23.6) | | <0.001 | | |
| Chronic rhinitis | 294 (19.3) | | 877 (19.3) | | 784 (17.1) | | 0.011 | | | 536 (16.7) | | 658 (17.1) | | 651 (18.5) | | 0.119 | | |
| Chronic rhinosinusitis | 67 ( 4.4) | | 136 ( 3.0) | | 106 ( 2.3) | | <0.001 | | | 65 ( 2.0) | | 59 ( 1.5) | | 69 ( 2.0) | | 0.236 | | |
|  | **Less than 30** | | | | | | | | **30- 45** | | | | | | | | |  |
|  | **Lowest** | **Upper lowe** | | **Lower high** | | **Highest** | | **P**  **value** | **Lowest** | | **Upper lowe** | | **Lower high** | | **Highest** | | **P**  **value** |  |
| n | 237 | 1830 | | 624 | | 1086 | |  | 311 | | 3613 | | 1609 | | 2842 | |  |  |
| Allergic rhinitis | 87  (36.7) | 646 (35.3) | | 210 (33.7) | | 368 (33.9) | | 0.722 | 101 (32.5) | | 1257 (34.8) | | 569 (35.4) | | 981 (34.5) | | 0.791 |  |
| Chronic rhinitis | 72  (30.4) | 471 (25.7) | | 145 (23.2) | | 233 (21.5) | | 0.008 | 72 (23.2) | | 756 (20.9) | | 281 (17.5) | | 480 (16.9) | | <0.001 |  |
| Chronic rhinosinusitis | 13  ( 5.5) | 67  ( 3.7) | | 18  ( 2.9) | | 36  ( 3.3) | | 0.303 | 15  ( 4.8) | | 137  ( 3.8) | | 36  ( 2.2) | | 69  ( 2.4) | | 0.001 |  |
|  | **46 - 60** | | | | | | | | **>=61** | | | | | | | | |  |
|  | **Lowest** | **Upper lowe** | | **Lower high** | | **Highest** | | **P**  **value** | **Lowest** | | **Upper lowe** | | **Lower high** | | **Highest** | | **P**  **value** |  |
| n | 308 | 4992 | | 1685 | | 2593 | |  | 210 | | 3770 | | 1268 | | 2110 | |  |  |
| Allergic rhinitis | 89  (28.9) | 1431 (28.7) | | 509 (30.2) | | 823 (31.7) | | 0.046 | 38 (18.1) | | 729 (19.3) | | 263 (20.7) | | 499 (23.6) | | 0.001 |  |
| Chronic rhinitis | 79  (25.6) | 911 (18.2) | | 329 (19.5) | | 419 (16.2) | | <0.001 | 42 (20.0) | | 680 (18.0) | | 248 (19.6) | | 378 (17.9) | | 0.546 |  |
| Chronic rhinosinusitis | 16  ( 5.2) | 144  ( 2.9) | | 36  ( 2.1) | | 49  ( 1.9) | | 0.001 | 3  ( 1.4) | | 76  ( 2.0) | | 17  ( 1.3) | | 30  ( 1.4) | | 0.234 |  |

Table S3. Prevalnce of forms of rhinitis by education and occupation levels within strata of those who were and were not raised on a farm during childhood.

|  | Raised on a farm | | | | | | Not raised on a farm | | | | | | | |  |  |  |  |
| --- | --- | --- | --- | --- | --- | --- | --- | --- | --- | --- | --- | --- | --- | --- | --- | --- | --- | --- |
|  | **Primary** | | **Secondary** | | **Tertiary** | | | **P**  **value** | | **Primary** | | **Secondary** | | **Tertiary** | | **P**  **value** | |  |
| n | 1358 | | 1724 | | 1424 | | |  | | 4173 | | 12278 | | 14592 | |  | |  |
| Allergic rhinitis | 220  (16.2) | | 360  (20.9) | | 343  (24.1) | | | <0.001 | | 856 (20.5) | | 3567  (29.1) | | 4762 (32.6) | | <0.001 | |  |
| Chronic rhinitis | 222  (16.3) | | 342  (19.8) | | 252  (17.7) | | | 0.039 | | 788 (18.9) | | 2542  (20.7) | | 2738 (18.8) | | <0.001 | |  |
| Chronic rhinosinusitis | 31  ( 2.3) | | 44  ( 2.6) | | 30  ( 2.1) | | | 0.705 | | 135  ( 3.2) | | 362  ( 2.9) | | 363  ( 2.5) | | 0.01 | |  |
|  | **Raised on a farm** | | | | | | | | **Not Raised on a farm** | | | | | | | | | |
|  | **Lowest** | **Upper lowe** | | **Lower high** | | **Highest** | **P**  **value** | | **Lowest** | | **Upper lowe** | | **Lower high** | | **Highest** | | **P**  **value** | |
| n | 169 | 2148 | | 469 | | 768 |  | | 875 | | 11903 | | 4658 | | 7748 | |  | |
| Allergic rhinitis | 44 (26.0) | 443 (20.6) | | 109 (23.2) | | 174 (22.7) | 0.221 | | 264 (30.2) | | 3578 (30.1) | | 1427 (30.6) | | 2462 (31.8) | | 0.085 | |
| Chronic rhinitis | 43 (25.4) | 386 (18.0) | | 107 (22.8) | | 126 (16.4) | 0.003 | | 219 (25.0) | | 2399 (20.2) | | 890 (19.1) | | 1368 (17.7) | | <0.001 | |
| Chronic rhinosinusitis | 9  ( 5.3) | 48  ( 2.2) | | 13  ( 2.8) | | 9  ( 1.2) | 0.007 | | 38  l ( 4.3) | | 368  ( 3.1) | | 93  ( 2.0) | | 175  ( 2.3) | | <0.001 | |

Table S4.Risk of forms of rhinitis by education and occupation skill levels as odds ratios and their 95% confidence intervals stratified by presence of asthma.

| Asthma | | | | | | | | | |
| --- | --- | --- | --- | --- | --- | --- | --- | --- | --- |
|  | Education | | | | Occupation | | | | |
| Allergic rhinitis | | | | | | | | | |
|  | **OR** | **Lower 95%** | **Upper 95%** | **P value** |  | **OR** | **Lower 95%** | **Upper 95%** | **P value** |
| **Primary education = Ref** | 1 |  |  |  | **Lowest = Ref** | 1 |  |  |  |
| **Secondary education** | 1.53 | 1.2 | 1.95 | 0.000 | **Upper low** | 1.13 | 0.73 | 1.73 | 0.58 |
| **Tertiary education** | 1.7 | 1.32 | 2.19 | 0.000 | **Lower high** | 1.23 | 0.76 | 1.97 | 0.4 |
|  |  |  |  |  | **Highest** | 1.08 | 0.68 | 1.68 | 0.74 |
| **Chronic rhinitis** | | | | | | | | | |
|  | **OR** | **Lower 95%** | **Upper 95%** | **P value** |  | **OR** | **Lower 95%** | **Upper 95%** | **P value** |
| **Primary education = Ref** | 1 |  |  |  | **Lowest = Ref** | 1 |  |  |  |
| **Secondary education** | 0.96 | 0.76 | 1.23 | 0.77 | **Upper low** | 0.87 | 0.58 | 1.31 | 0.51 |
| **Tertiary education** | 0.84 | 0.65 | 1.07 | 0.16 | **Lower high** | 0.86 | 0.55 | 1.35 | 0.5 |
|  |  |  |  |  | **Highest** | 0.87 | 0.57 | 1.34 | 0.52 |
| **Chronci rhinosinusits** | | | | | | | | | |
|  | **OR** | **Lower 95%** | **Upper 95%** | **P value** |  | **OR** | **Lower 95%** | **Upper 95%** | **P value** |
| **Primary education = Ref** | 1 |  |  |  | **Lowest = Ref** | 1 |  |  |  |
| **Secondary education** | 0.71 | 0.49 | 1.04 | 0.07 | **Upper low** | 0.79 | 0.44 | 1.51 | 0.44 |
| **Tertiary education** | 0.74 | 0.5 | 1.12 | 0.15 | **Lower high** | 0.55 | 0.26 | 1.2 | 0.13 |
|  |  |  |  |  | **Highest** | 1.21 | 0.64 | 2.4 | 0.58 |
| **No asthma** | | | | | | | | | |
| Allergic rhinitis | | | | | | | | | |
|  | **OR** | **Lower 95%** | **Upper 95%** | **P value** |  | **OR** | **Lower 95%** | **Upper 95%** | **P value** |
| **Primary education = Ref** | 1 |  |  |  | **Lowest = Ref** | 1 |  |  |  |
| **Secondary education** | 1.34 | 1.22 | 1.48 | 0.000 | **Upper low** | 1.04 | 0.87 | 1.24 | 0.690 |
| **Tertiary education** | 1.59 | 1.44 | 1.75 | 0.000 | **Lower high** | 1.17 | 0.97 | 1.41 | 0.100 |
|  |  |  |  |  | **Highest** | 1.17 | 0.98 | 1.4 | 0.090 |
| **Chronic rhinitis** | | | | | | | | | |
|  | **OR** | **Lower 95%** | **Upper 95%** | **P value** |  | **OR** | **Lower 95%** | **Upper 95%** | **P value** |
| **Primary education = Ref** | 1 |  |  |  | **Lowest = Ref** | 1 |  |  |  |
| **Secondary education** | 1.21 | 1.1 | 1.34 | 0.000 | **Upper low** | 0.88 | 0.73 | 1.06 | 0.160 |
| **Tertiary education** | 1.23 | 1.11 | 1.36 | 0.000 | **Lower high** | 1.06 | 0.87 | 1.29 | 0.570 |
|  |  |  |  |  | **Highest** | 0.95 | 0.79 | 1.15 | 0.580 |
| **chronic rhinosinusitis** | | | | | | | | | |
|  | **OR** | **Lower 95%** | **Upper 95%** | **P value** |  | **OR** | **Lower 95%** | **Upper 95%** | **P value** |
| **Primary education = Ref** | 1 |  |  |  | **Lowest = Ref** | 1 |  |  |  |
| **Secondary education** | 0.97 | 0.75 | 1.26 | 0.820 | **Upper low** | 0.94 | 0.62 | 1.49 | 0.780 |
| **Tertiary education** | 1.01 | 0.77 | 1.34 | 0.940 | **Lower high** | 1.03 | 0.65 | 1.71 | 0.900 |
|  |  |  |  |  | **Highest** | 0.9 | 0.57 | 1.46 | 0.650 |

Table S5. Risk of forms of rhinitis by education and occupation skill levels as odds ratios and their 95% confidence intervals stratified by urban versus childhood residence.

| Rural childhood | | | | | | | | | |
| --- | --- | --- | --- | --- | --- | --- | --- | --- | --- |
| Education | | | | | Occupation | | | | |
| allergic rhinitis | | | | | | | | | |
|  | **OR** | **Lower 95%** | **Upper 95%** | **P value** |  | **OR** | **Lower 95%** | **Upper 95%** | **P value** |
| Primary education = Ref | 1 |  |  | 0 | Lowest = Ref |  |  |  |  |
| Secondary education | 1.31 | 1.14 | 1.5 | 0 | Upper low | 1.04 | 0.8 | 1.35 | 0.79 |
| Tertiary education | 1.51 | 1.31 | 1.74 | 0 | Lower high | 1.21 | 0.92 | 1.61 | 0.19 |
|  |  |  |  |  | Highest | 1.21 | 0.93 | 1.6 | 0.16 |
| **chronic rhinitis** | | | | | | | | | |
|  | **OR** | **Lower 95%** | **Upper 95%** | **P value** |  | **OR** | **Lower 95%** | **Upper 95%** | **P value** |
| Primary education = Ref | 1 |  |  |  | Lowest = Ref | 1 |  |  |  |
| Secondary education | 1.2 | 1.04 | 1.38 | 0.01 | Upper low | 0.76 | 0.58 | 0.99 | 0.04 |
| Tertiary education | 1.24 | 1.07 | 1.44 | 0 | Lower high | 0.98 | 0.74 | 1.3 | 0.87 |
|  |  |  |  |  | Highest | 0.92 | 0.7 | 1.22 | 0.57 |
| **Chronic rhinosinusitis** | | | | | | | | | |
|  | **OR** | **Lower 95%** | **Upper 95%** | **P value** |  | **OR** | **Lower 95%** | **Upper 95%** | **P value** |
| Primary education = Ref | 1 |  |  |  | Lowest = Ref | 1 |  |  |  |
| Secondary education | 0.92 | 0.66 | 1.3 | 0.64 | Upper low | 0.83 | 0.48 | 1.51 | 0.51 |
| Tertiary education | 1.07 | 0.74 | 1.54 | 0.73 | Lower high | 0.95 | 0.51 | 1.85 | 0.87 |
|  |  |  |  |  | Highest | 0.94 | 0.52 | 1.79 | 0.83 |
| **Urban childhood** | | | | | | | | | |
| **Allergic rhinitis** | | | | | | | | | |
|  | **OR** | **Lower 95%** | **Upper 95%** | **P value** |  | **OR** | **Lower 95%** | **Upper 95%** | **P value** |
| Primary education = Ref | 1 |  |  |  | Lowest = Ref | 1 |  |  |  |
| Secondary education | 1.38 | 1.23 | 1.54 | 0 | Upper low | 1.06 | 0.87 | 1.3 | 0.56 |
| Tertiary education | 1.6 | 1.43 | 1.8 | 0 | Lower high | 1.12 | 0.91 | 1.38 | 0.29 |
|  |  |  |  |  | Highest | 1.14 | 0.93 | 1.4 | 0.2 |
| **Chronic rhinitis** | | | | | | | | | |
|  | **OR** | **Lower 95%** | **Upper 95%** | **P value** |  | **OR** | **Lower 95%** | **Upper 95%** | **P value** |
| Primary education = Ref | 1 |  |  |  | Lowest = Ref | 1 |  |  |  |
| Secondary education | 1.18 | 1.05 | 1.33 | 0.01 | Upper low | 0.96 | 0.78 | 1.19 | 0.71 |
| Tertiary education | 1.15 | 1.02 | 1.3 | 0.03 | Lower high | 1.04 | 0.83 | 1.31 | 0.73 |
|  |  |  |  |  | Highest | 0.96 | 0.77 | 1.19 | 0.69 |
| **Chronic rhinosinusitis** | | | | | | | | | |
|  | **OR** | **Lower 95%** | **Upper 95%** | **P value** |  | **OR** | **Lower 95%** | **Upper 95%** | **P value** |
| Primary education = Ref | 1 |  |  |  | Lowest = Ref | 1 |  |  |  |
| Secondary education | 0.89 | 0.68 | 1.18 | 0.41 | Upper low | 0.92 | 0.6 | 1.48 | 0.72 |
| Tertiary education | 0.89 | 0.67 | 1.18 | 0.4 | Lower high | 0.8 | 0.48 | 1.35 | 0.38 |
|  |  |  |  |  | Highest | 1.02 | 0.65 | 1.68 | 0.93 |

Table S6. Results of gender- and age-modified association between education and allergic rhinitis.

|  | Sex | Odds. ratio | Lower 95% | Upper 95% |
| --- | --- | --- | --- | --- |
| Allergic rhinitis | | | | |
| Secondary education / Primary education | Male | 1.53 | 1.30 | 1.81 |
| Tertiary education / Primary education | Male | 2.02 | 1.71 | 2.38 |
| Tertiary education / Secondary education | Male | 1.32 | 1.18 | 1.47 |
| Secondary education / Primary education | Female | 1.36 | 1.16 | 1.61 |
| Tertiary education / Primary education | Female | 1.56 | 1.33 | 1.83 |
| Tertiary education / Secondary education | Female | 1.14 | 1.04 | 1.26 |
|  | **Age groups** | **Odds. ratio** | **Lower 95%** | **Upper 95%** |
| Chronic rhinitis | | | | |
| Secondary education / Primary education | less than 30 | 1.02 | 0.75 | 1.39 |
| Tertiary education / Primary education | less than 30 | 0.92 | 0.68 | 1.25 |
| Tertiary education / Secondary education | less than 30 | 0.90 | 0.77 | 1.06 |
| Secondary education / Primary education | 30-45 | 1.12 | 0.82 | 1.54 |
| Tertiary education / Primary education | 30-45 | 1.03 | 0.75 | 1.40 |
| Tertiary education / Secondary education | 30-45 | 0.92 | 0.79 | 1.05 |
| Secondary education / Primary education | 46-60 | 1.18 | 0.97 | 1.44 |
| Tertiary education / Primary education | 46-60 | 1.18 | 0.96 | 1.44 |
| Tertiary education / Secondary education | 46-60 | 1.00 | 0.87 | 1.15 |
| Secondary education / Primary education | >=61 | 1.14 | 0.96 | 1.35 |
| Tertiary education / Primary education | >=61 | 1.39 | 1.18 | 1.65 |
| Tertiary education / Secondary education | >=61 | 1.22 | 1.05 | 1.43 |

Table S7. Results of body mass index -modified association between education and chronic rhinitis.

|  | BMI | odds. Ratio | Lower 95% | Upper 95% |
| --- | --- | --- | --- | --- |
| Secondary education / Primary education | Normal weight | 1.11 | 0.93 | 1.31 |
| Tertiary education / Primary education | Normal weight | 1.03 | 0.87 | 1.22 |
| Tertiary education / Secondary education | Normal weight | 0.93 | 0.83 | 1.03 |
| Secondary education / Primary education | Overweight | 1.23 | 1.04 | 1.47 |
| Tertiary education / Primary education | Overweight | 1.33 | 1.11 | 1.58 |
| Tertiary education / Secondary education | Overweight | 1.08 | 0.95 | 1.22 |
| Secondary education / Primary education | Obese | 1.17 | 0.93 | 1.47 |
| Tertiary education / Primary education | Obese | 1.24 | 0.98 | 1.58 |
| Tertiary education / Secondary education | Obese | 1.06 | 0.88 | 1.28 |


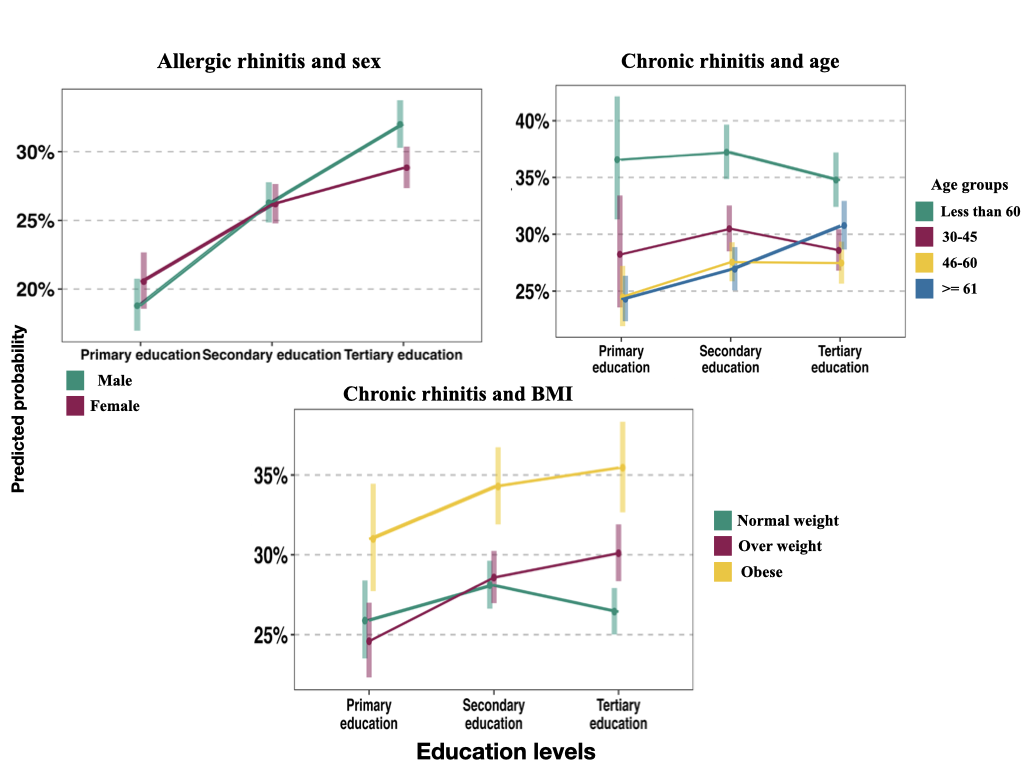


Figure S1. Interaction between education and baseline characteristics towards risk of forms of rhinitis
